# Etiology of super-enhancer reprogramming and activation in cancer

**DOI:** 10.1186/s13072-023-00502-w

**Published:** 2023-07-06

**Authors:** Royce W. Zhou, Ramon E. Parsons

**Affiliations:** 1 The Tisch Cancer Institute, Icahn School of Medicine at Mount Sinai, New York, NY USA; 2 Department of Oncological Sciences, Icahn School of Medicine at Mount Sinai, New York, NY USA; 3 Molecular Medicine Program, University of California San Francisco Internal Medicine Residency, San Francisco, CA, USA

**Keywords:** Enhancers, Super-enhancers, Cancer, Extrachromosomal DNA, Topologically associated domain, Non-coding mutations, Phase separation, Inflammation, Therapeutic resistance, Insulators, Tumor microenvironment

## Abstract

Super-enhancers are large, densely concentrated swaths of enhancers that regulate genes critical for cell identity. Tumorigenesis is accompanied by changes in the super-enhancer landscape. These aberrant super-enhancers commonly form to activate proto-oncogenes, or other genes upon which cancer cells depend, that initiate tumorigenesis, promote tumor proliferation, and increase the fitness of cancer cells to survive in the tumor microenvironment. These include well-recognized master regulators of proliferation in the setting of cancer, such as the transcription factor *MYC* which is under the control of numerous super-enhancers gained in cancer compared to normal tissues. This Review will cover the expanding cell-intrinsic and cell-extrinsic etiology of these super-enhancer changes in cancer, including somatic mutations, copy number variation, fusion events, extrachromosomal DNA, and 3D chromatin architecture, as well as those activated by inflammation, extra-cellular signaling, and the tumor microenvironment.

## Background

Super-enhancers are estimated to contain tenfold more distinct protein factors than typical enhancers and are algorithmically defined using the Rank Ordering of Super-Enhancers (ROSE) script [1–3]. Using chromatin immunoprecipitation of active regulatory marks, most commonly Med1, BRD4, or H3K27ac coupled with next-generation sequencing (ChIP-seq), ROSE identifies areas of the epigenome with exceptionally high signal density known as super-enhancers (SEs).

SEs were first discovered in transgenic mice in and termed locus control regions in 1987. Further characterized in murine embryonic stem cells (mESCs), super-enhancers adopted their moniker in 2013 and were observed near transcription factors required for pluripotency, suggesting they may enrich at genes critical for cell identity in the setting of healthy and diseased states [4, 5]. Several genes involved in tumorigenesis and tumor progression were similarly found to be activated by SEs in cancer, notably *MYC* [2]*.* Transcriptional regulation by SEs is often critical for downstream gene expression, as CRISPR deletion or interference of the distal SE significantly reduces expression of its target gene. In the case *MYC,* deletion of its SE in mice results in complete loss of *MYC* expression in hematopoietic lineages [6].

The etiologies of enhancer changes described in cancer are almost invariably attributed to cell-intrinsic genomic alterations, including (1) activation of oncogenic signaling and, (2) de novo formation of transcription factor (TF) binding sites, as well as (3) focal amplification of non-coding active regulatory regions [7–10]. Here, we highlight literature on the origins of enhancer and SE reprogramming in cancer, including recent advances in cell-extrinsic SE reprogramming by the tumor microenvironment as well as in extrachromosomal DNA, phase separation, and higher order chromatin structure.

### Oncogenic signaling

**Fig. 1:**
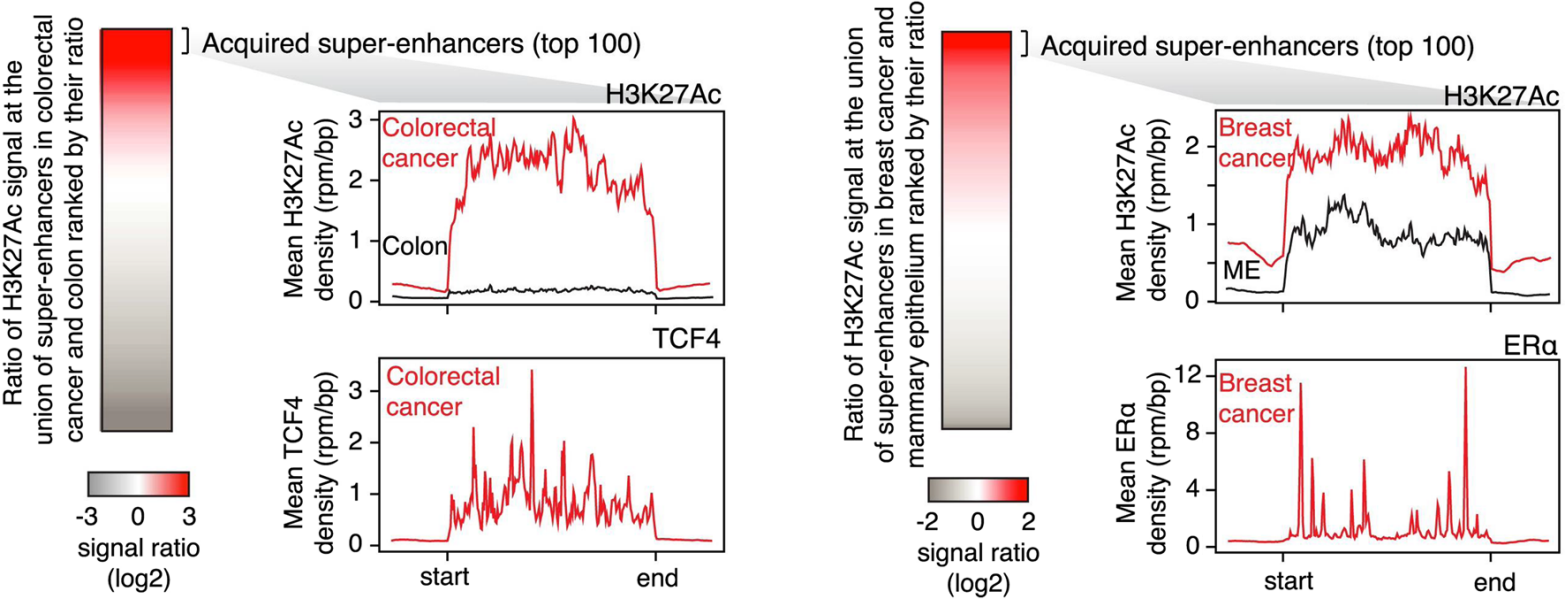
Oncogenic signaling results in transcription factor occupancy at gained SEs in cancer. Metagene plots showing TCF4 occupancy (red, left bottom), a transcription factor activated by Wnt signaling, at SEs gained in CRC over normal colon (left top). Metagenes plots showing ERα occupancy (red, right bottom) at SEs gained in ER+ breast cancer over normal breast (right top). Figure reproduced with permission from Elsevier. Please refer to the original publication (Hnisz et al. [7]) for more details and citation

Perhaps the most well-recognized etiology of SE reprogramming in cancer is downstream of somatic coding region mutations that activate oncogenes or inactivate tumor suppressor genes. In renal cell carcinoma, loss of the frequently mutated tumor suppressor *VHL* that encodes the E3 ligase for the HIF transcription factor, directly results in the formation of numerous aberrant SEs due to HIF accumulation [11]. In sporadic colorectal cancers, ~ 80% of cases are observed to have mutations in the tumor suppressor *APC* that encodes a member of the β-catenin destruction complex [12]*.* Accumulated β-catenin subsequently translocates to the nucleus to bind the TCF/LEF family of transcription factors and activate transcription of Wnt target genes including the previously mentioned and known target *MYC* (Fig. 1) [13]. Indeed, Hnisz et al. observed dense binding of TCF4 within the *MYC* SE and its motif was enriched among gained SEs in CRC [7]*.* Thus, oncogenic signaling from somatic mutations shape the super-enhancer landscape in CRC [7].

Gain-of-function point mutations in transcription factors can also cause aberrant SE formation. The KLF5 transcription factor, an oncogene implicated in several cancers including CRC, exhibits hotspot E419Q mutations in its DNA binding domain results in over 5000 gained binding sites compared to WT KLF5 including de novo SEs at pro-tumorigenic genes [14]. In line with this finding, lung cancer cells overexpressing KLF5 E419Q exhibited greater proliferation than WT. Transcription factor mutations that create SEs are not exclusive to the DNA binding domain. In lymphoma, the frequently mutated transcriptional activator MEF2B exhibits N-terminal hotspot mutations at regulatory residues [15]. These allow the MEF2B D38V mutant to evades repressor binding and, in turn, bind lymphoma-promoting genes [16].

Inactivating mutations in proteins that directly modify histones can also reprogram SEs, especially proteins governing histone methylation, which are mutated in a variety of cancers [17]. Loss of histone methyltransferase *KMT2D* (also known as *MLL4*) in lung cancer results in a global reduction in SE acetylation levels due to inhibition of H3K4 methylation at promoters, including at the transcription factor *PER2* which negatively regulates glycolytic genes [18]. Yet, disinhibition of these *PER2-*dependent glycolytic genes nevertheless sufficiently reprograms the SE landscape of these genes to sustain a metabolic dependency in this subset of lung cancer with increased sensitivity to glycolysis inhibiton [18]. Furthermore, loss of MLL4 also impairs formation of de novo oncogenic SEs by the aberrant expression of HOXA9 transcription factor in acute leukemia [19].

In several cancers, whole genome and whole exome sequencing approaches uncovered high frequency mutations in the SWI/SNF family of chromatin modifying enzymes. The SWI/SNF complex features ATP-ase activity capable of de-stabilizing histone–DNA interactions and thereby regulating chromatin accessibility for transcription factor binding. Thus, mutations in the SWI/SNF family, collectively observed in ~ 20% of all human cancers, can impact the enhancer landscape to varying degrees [20]. The discrepancy in effects is likely dependent on which complex member is lost and evicted and the subsequent specific activity of the remaining complex, as well as the tissue-specific chromatin state it operates within.

For instance, *PBRM1*, which encodes the BAF180 subunit of SWI/SNF, is frequently lost in renal cell carcinoma (RCC). However, *PBRM1* silenced RCC cell lines exhibit little change in open chromatin and H3K27ac landscapes including at SEs [21]. Similarly, almost all pediatric rhabdoid tumors exhibit loss of SWI/SNF core subunit *SMARCB1,* which encodes the subunit SNF5*. SMARCB1* loss in this context decreases SWI/SNF occupancy at typical enhancers (TEs) while maintaining occupancy at SEs, an observation that was reproduced in other cancer cell lines as well [22–24].

Conversely, a separate study showed loss of *ARID1A,* mutated in endometrial carcinoma, preferentially affects SEs over TEs. Wilson et al. observed ARID1A occupancy at SEs over TEs, which exhibited increased H3K27ac signal and open chromatin accessibility following *ARID1A* deletion resulting in activation of invasion genes [25]. These sites were most significantly co-enriched with the histone acetyl-transferase (HAT) P300, which has known roles in enhancer and SE regulation [25]. Epistasis experiments show that both the hyperacetylation of select SEs and the greater invasive phenotype in endometrial carcinoma observed upon *ARID1A* deletion is attenuated with either genetic or pharmacologic inhibition of P300, suggesting it is required in the setting of *ARID1A* loss [25]. The exact mechanism of ARID1A and P300 interplay is not fully understood, but appears to be independent of P300 recruitment as *ARID1A* deletion did not change significantly change P300 genome-wide occupancy [25]. A follow-up study proposes an alternative mechanism. ChIP-seq studies revealed significant co-localization of *ARID1A* and the repressive histone variant H3.3, which became depleted following *ARID1A* loss [26].

### Fusion proteins and phase separation

**Fig. 2:**
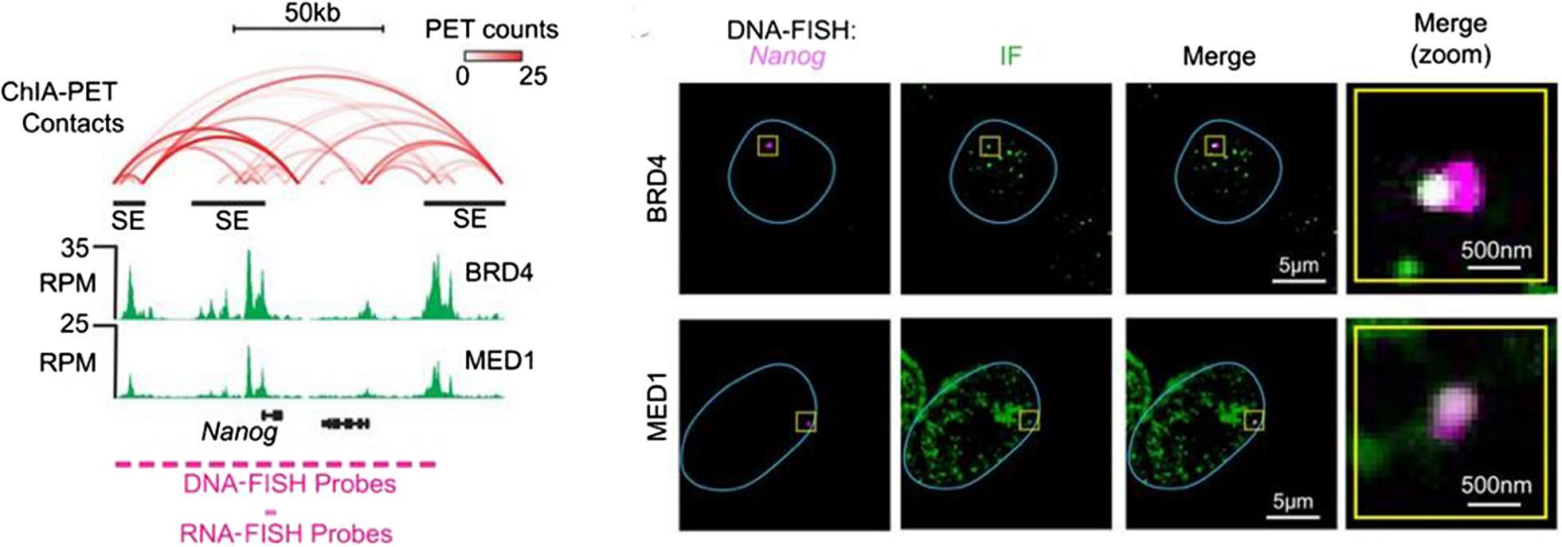
Super-enhancers exist as Phase separated liquid condensates. DNA-Fluorescent in situ Hybridization (DNA–FISH) of the *Nanog* SE shows punctate staining in the mouse embryonic stem cells. Co-immunofluorescence (IF) of super-enhancer transcriptional machinery proteins BRD4 and MED1 also show a punctate staining pattern, suggestive of phase separated liquid condensates. Merged view shows overlap of *Nanog* DNA–FISH and BRD4 and MED1 IF puncta, suggesting co-occupancy of the same liquid condensate. Figure reproduced with permission from Science. Please refer to the original publication (Sabari et al. [40]) for more details and citation

The proximity of constituent enhancers, the density of protein factors, and the level of transcriptional cooperativity led to the hypothesis that SEs exist as membrane-less, phase separated condensates, which have recently emerged as an important driver of protein–protein interactions, especially given the intrinsically disordered domains present among transcription factors, Mediator, and BRD4 (Fig. 2) [3, 32–39]. Indeed, in vitro assays of GFP-tagged intrinsically disordered domains alone, derived from BRD4 or Med1, show condensate formation [40]. The significance of this discovery was in uncovering a structural basis for enabling SE control of gene expression. Liquid condensates exhibit unique aggregation and dispersion properties that appear to specifically associated with SEs over typical enhancers. Microscopy showed DNA–FISH probes against SEs, Med1, and BRD4 to exist as overlapping puncta that could be dispersed with 1,6-hexanediol, which disrupts liquid condensates (Fig. 2) [35, 37, 38, 40]. Notably, puncta dispersion correlated with loss of Med1, BRD4, and RNA polymerase II at SEs. Furthermore, MED1 partitioning recruits RNA polymerase II and its positive regulators while excluding negative regulators [41]. Recent biochemical assays show that this phenomenon, termed selective partitioning, depends on the charge pattern of residues within intrinsically disordered regions [41]. Surprisingly, in vitro droplet assays demonstrate cytotoxic chemotherapy agents, such as cisplatin, also congregate within condensates, with a preference for Med1 [42, 43]. This new area of study provides new mechanistic leads for the selective targeting of SE gene expression in cancer, a sought-after goal since the discovery of SEs.

Oncogenic fusion proteins that create aberrant transcription factors can create de novo SEs to maintain transcriptional dependencies. One recurrently detected fusion in leukemia, NUP98–HOXA9, is enriched for intrinsically disordered domains to form a *de novo* liquid–liquid phase separated puncta with subsequent oncogenic SE formation detected by ChIP-seq [44, 45]. Notably, fusion proteins with fewer phenylalanine and glycine repeats attenuated phase separation, as did mutating phenylalanine residues to serine [44]. Importantly, these changes also decreased leukemia transformation [44]. Other oncogenic fusion proteins capable of forming and sustaining aberrant SEs include PAX3–FOXO1 in rhabdomyosarcoma, ZFTA–RELA in ependymoma, ETO2–GLIS2 in acute megakaryoblastic leukemia, and TCF3–HLF in acute lymphoblastic leukemia [27–30]. SEs themselves may even be stitched together in an oncogenic fusion event to form a large hybrid SE, such as C19MC–TTYH1 in embryonal tumors with multi-layered rosettes to promote C19MC onco-miRNA expression [31].

### Non-coding mutations and polymorphisms

**Fig. 3:**
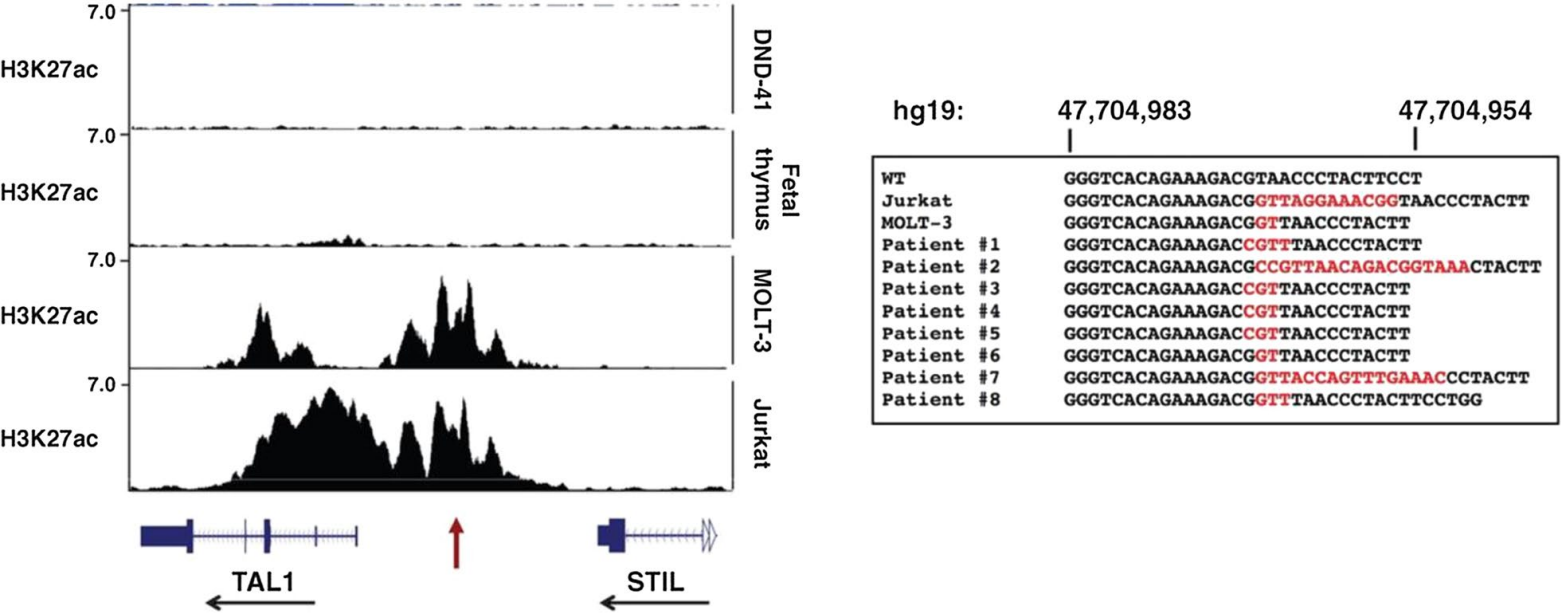
Recurrent non-coding insertions lead to super-enhancer formation and *TAL1* expression. Left, H3K27ac ChIP-seq tracks showing a super-enhancer at the locus of *TAL1* in Jurkat and MOLT-3 cells, both of T-cell acute lymphoblastic leukemia (T-ALL) origin. This super-enhancer is absent in DND-41 T-cell leukemia cells and fetal thymus cells. Right, non-coding region sequences underlying the *TAL1* super-enhancer. Note the recurrent insertions (in red) in Jurkat and MOLT-3 cells, as well as in eight patients with T-ALL, that correlate with formation of the *TAL1* super-enhancer. Figure reproduced with permission from Science. Please refer to the original publication (Mansour et al. [8]) for more details and citation

Mutations in non-coding regions of the genome are observed in cancer. While most are believed to be passenger events, a few are functionally relevant to cancer cells. Mansour et al. describe a SE in T-ALL that overlapped a recurrent somatic insertion in an intergenic region [8]. This insertion creates a *de novo* binding site for the transcription factor MYB that resulted in the formation of a SE at this locus (Fig. 3) [7]. The downstream gene, *TAL1,* was rendered exquisitely sensitive to *MYB* silencing, suggesting it assumes key regulatory control [8]*.* As proof-of-principle, CRISPR-cas9 deletion of the somatic insertion collapses the super-enhancer, reduces *TAL1* expression, and affects cell viability—one of the first studies to concretely demonstrate somatic mutations in non-coding regions could form enhancers that are inherently oncogenic [8].

A more systematic process of non-coding somatic mutations occurs in B cell lymphomas. B cells express activation-induced cytidine deaminase (AID) for somatic hypermutation and class switch recombination [46, 47]. However, these can occur at non-immunologic loci that generate translocations and mutations that contribute to B cell lymphoma tumorigenesis. A recent study showed 92% of diffuse large B cell lymphoma (DLBCL) samples, the most common form of lymphoma in the US, exhibit hypermutation and a characteristic AID mutation signature [48]. In 2014, two studies identified AID activity at B cell SEs, with subsequent hypermutation of these non-coding SE regions, including hotspot mutations suggestive of selection at the SE regulating *BCL6,* a transcription factor that regulates B cell proliferation [46–49].

In contrast to the Mansour et al. study in T-ALL in which a mutation created a binding site for a transcription factor to form a SE, non-coding mutations in DLBCL occur in existing SEs and alter the binding sequences of transcriptional repressors. This disinhibition results in even greater expression of SE associated oncogenes including *BCL6, BCL2,* and *CXCR4* [48]. At the *BCL6* SE, a recurrent mutation prevents binding by the transcriptional repressor BLIMP1 and confers increased fitness in DLBCL cells. When these mutations were corrected back to the WT allele, dropout of DLBCL cells was observed compared to isogenic cells retaining the *BCL6* SE mutation [48]. These data show how the process of somatic hypermutation in B cells amplify SE-mediated oncogene expression. 

Inborn polymorphisms that affect transcription factor binding also occur in SEs. Oldridge et al. report a G > T polymorphism at the SE of the transcriptional co-regulator and oncogene *LMO1* that associated with neuroblastoma susceptibility in GWAS [50]*.* The G is the reference and risk allele, critical for the GATAA binding motif for the GATA transcription factor within the SE, while the protective alternative allele T breaks this sequence, resulting in decreased GATA occupancy at the *LMO1* SE and gene expression [50]. In a European cohort, heterozygous (G/T) and homozygous (T/T) protective allele carriers exhibited significantly increased survival compared to G/G patients [50]. A similar study showed a C > T somatic mutation at an enhancer region of *LMO1*, suggested to originate from an APOBEC-like cytidine deaminase mutational signature, that created a MYB transcription factor binding site which increased expression and dependency on *LMO1* in T-ALL [51]. However, this enhancer was not of sufficient size or density to meet SE criteria [51]. These examples provide further examples of how single nucleotide variations can create or delete transcription factor binding motifs with consequences on SE formation, downstream gene expression, and cancer dependency.

### Focal amplification

Amplification is one of the most established etiologies of SE reprogramming in cancer alongside oncogenic signaling. Approximately 25% of neuroblastomas exhibit amplification of the oncogene *MYCN* which is the strongest correlate for high-risk disease and poor prognosis [52]. While non-amplified MYCN binds to a canonical CACGTG sequence, amplified *MYCN* results in widespread promiscuous occupation of an ubiquitous CANNTG motif (where N can represent any base) [53]. The result is amplified MYCN binding nearly every promoter and enhancer in the genome in a phenomenon termed “enhancer invasion” that results in globally increased transcription [53]. Dysregulated MYCN is sufficient to form aberrant oncogenic SEs in neuroblastoma [54].

**Fig. 4:**
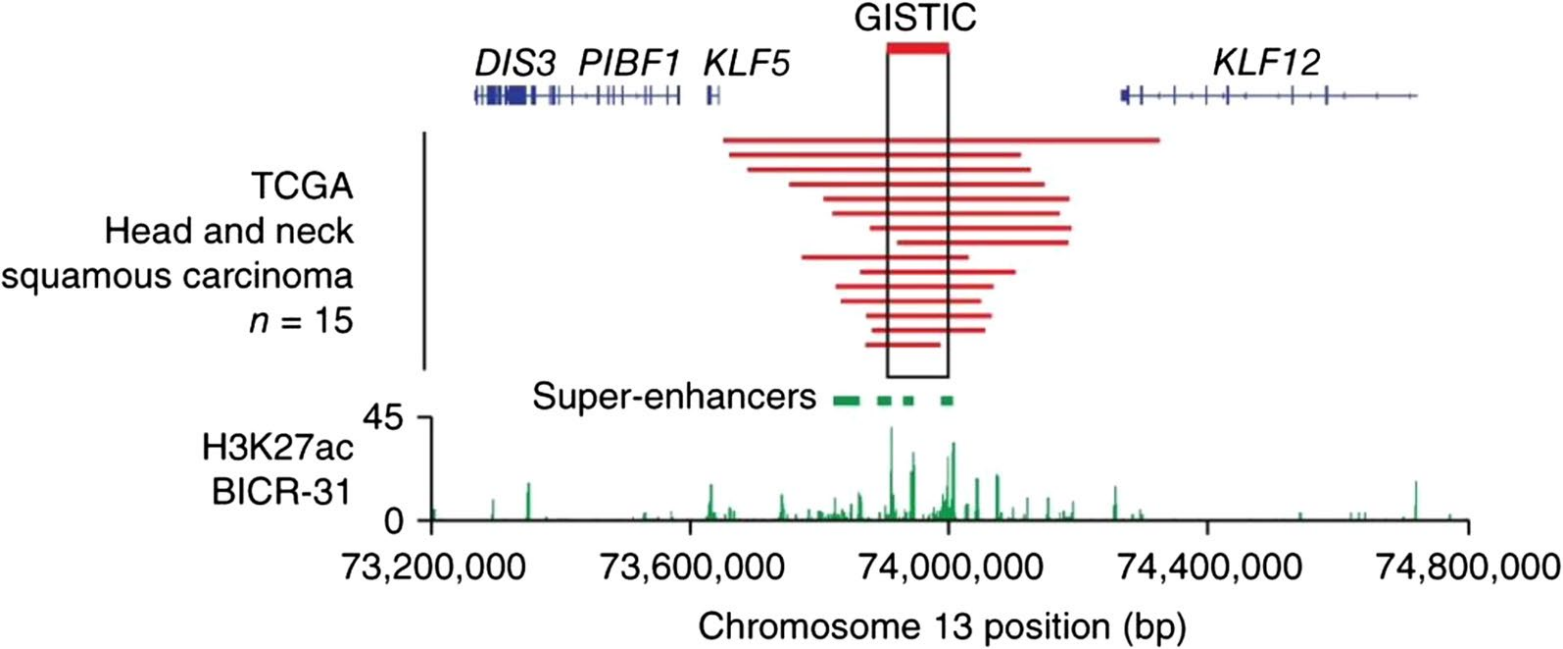
Recurrent focal amplification of a super-enhancer at the *KLF5* locus. Recurrent focal amplification (red) in 15 patients with head and neck squamous cell carcinoma (HNSCC) from TCGA in a non-coding region near *KLF5.* Beneath, H3K27ac ChIP-seq tracks from the HNSCC cell line, BICR-31, with called super-enhancers annotated by green bars. Note the overlap between amplified regions and SEs near *KLF5.* Figure reproduced with permission from Nature Genetics. Please refer to the original publication (Zhang et al. [9]) for more details and citation

Non-coding regions containing super-enhancers may similarly be amplified over the course of tumorigenesis [55–59]. An early study performed by the laboratory of Matthew Meyerson analyzed cancer cases from TCGA in search of recurrently amplified non-coding regions that overlapped with SEs [9]. In line with multiple reports, Zhang et al. found multiple recurrently amplified SEs at the loci for *MYC, KLF5, USP12,* and *PARD6B* (Fig. 4) [9]. Interestingly, the region around *MYC* that was amplified differed among different cancer types, suggesting tissue or lineage specific dysregulation [9]*.* The TCGA ATAC-seq study performed by the laboratory of Howard Chang similarly noted different open chromatin patterns at the *MYC* locus in different cancers [60]. A follow-up study showed the *KLF5* SE is focally amplified in numerous different cancers, albeit at low frequency, with increased dependency of cancer cells on *KLF5* expression [14]. Similar observations in breast cancer showed tandem duplication of SE regions that activate oncogenes, including *ESR1, ZNF217,* and *MYC* [57]. These studies demonstrate focal amplification of non-coding regions as an origin of SE reprogramming in cancer.

### Extrachromosomal DNA

By a separate mechanism, high-level amplification of oncogenes may arise from chromothripsis that result in amplicons carried on extrachromosomal DNA (ecDNA) ranging from 100 kb to several megabases, a process linked to p53 mutations [10, 61–64]. These circular pieces of DNA lack centromeres and randomly segregate into daughter cells during cell division with tens to hundreds of copies per cell [63, 65, 66]. The heterogeneity and plasticity of ecDNA rapidly confer fitness to cancer cells, including resistance to therapy [63, 67]. Furthermore, they do not exhibit the level of compartmentalization observed on chromosomal DNA, which may explain their highly accessible chromatin [68].

Using *EGFR* as an example, Morton et al. show enhancers potentiating *EGFR* expression are co-amplified on the same amplicon as the oncogene on ecDNA, confirmed using DNA fluorescence in situ hybridization (FISH) [10, 66]. This observation extends to the co-amplification of SEs with *MYCN* in neuroblastoma and Wilms tumor [10]. DNA breaks from chromothripsis are believed to occur and re-assemble at random during repair [69]. Remarkably, Morton et al. observed enhancers on non-contiguous regions of DNA occupying different topologically associated domains (TADs), assembled in a single compact ecDNA amplicon [10, 70]. Complex rearrangements may thus synthetically pair distal enhancers and genes in *cis* on ecDNA [68].

SE-gene interactions are not restricted to just ecDNAs. One study found that SEs on ecDNA can interact with and enhance expression of genes on chromosomal DNA, thus acting as “mobile enhancers” [71]. ecDNA can also congregate as hubs within the nucleus of cancer cells, a structural orientation that functionally affects downstream expression [72, 73]. These hubs appear to require bromodomains, as they co-localize with epitope labeled BRD4 and scatter upon treatment with bromodomain inhibitors, which also prevent their re-assembly following mitosis [73]. Interestingly, analyses suggest ecDNAs within hubs are more transcriptionally active than “singleton” non-hub associated ecDNAs, including at *MYC* [66, 73]*.* Hung et al. propose a model, where enhancer activation of the *MYC* promoter can occur in *cis,* from an enhancer within the same ecDNA, or in *trans,* from an enhancer on another ecDNA molecule within the same hub [73]. The phenomenon appears to be sequence specific or under additional layers of regulation. Indeed, in a gastric cancer cell line, where *FGFR2* ecDNA and *MYC* ecDNA are in a hub, interference of *FGFR2* enhancers decreased *MYC* expression, but interference of *MYC* enhancers did not affect *FGFR2* expression [73]. The implication of this finding is that enhancers from different chromosomes, *MYC* on chromosome 8 and *FGFR2* on chromosome 10, can activate genes in *trans* through ecDNAs*.*

A technical difficulty of ecDNA studies have been trying to distinguish between chromosomal versus extrachromosomal reads in sequencing experiments. Recently, Hung et al. from the laboratory of Howard Chang developed CRISPR–CATCH, a novel technique for the isolation of megabase-scale ecDNA that allows targeted investigation of the epigenome within these amplicons [74]. Indeed, 60% of sequenced reads from CRISPR–CATCH isolated DNA was posited to be extrachromosomal, compared to 2% from whole cellular DNA [74]. We anticipate this technique to elucidate further structural and epigenetic insight into this emerging form of transcriptional dysregulation in cancer.

### Topologically associated domains and insulator boundaries

SEs are often flanked by CTCF sites with exceptionally strong boundaries believed to concentrate transcriptional activating machinery at a focal locus to potentiate gene expression [75]. These strong boundaries are observed to be co-duplicated alongside SEs in cancer patients, suggesting structural significance in maintaining oncogene expression [75].

DNA between two CTCF sites are extruded to form a loop, compartmentalizing enhancers and gene bodies into “neighborhoods” or topologically associating domains (TADs) thereby restricting gene–enhancer interactions to mainly within the loop. However, complex, long-distance SE interactions have also been described, including three-way interactions between multiple SE-containing TADs on the same chromosome [76]. Perturbing a CTCF boundary can merge two relatively insulated TADs into one, with consequences for gene–enhancer interactions [77–79].

Flavahan et al. showed, in two studies, that such disruption can unleash SEs to activate nearby oncogenes. One such mechanism of CTCF perturbation is methylation, a feature of several cancer especially those with mutations in isocitrate dehydrogenase, *IDH* [80–83]. These are commonly gain-of-function hotspot mutations in the isocitrate binding site that confer a neomorphic ability to convert α-ketoglutarate to millimolar concentrations of 2-hydroxyglutarte (2-HG) which directly inhibit DNA and histone demethylases resulting in hypermethylation, disruption of TADs, and altered expression [84].

**Fig. 5:**
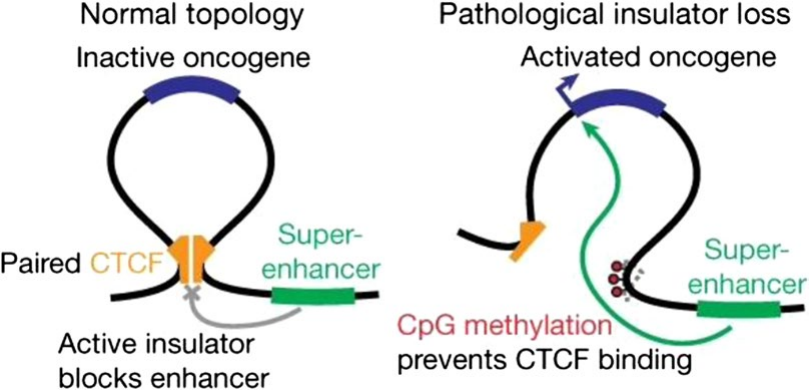
CTCF boundaries restrain super-enhancers from aberrant oncogene activation. Left, under physiologic conditions, a CTCF insulator separates a super-enhancer from a neighboring oncogene and prevents SE–promoter interactions that would lead to oncogene expression. Right, loss of CTCF binding from methylation results in boundary disruption that permits SE–oncogene interactions that result in activation. Figure reproduced with permission from Nature. Please refer to the original publication (Flavahan et al. [91]) for more details and citation

*PDGFRA* amplifications and mutations are common in glioblastoma but appear to be somewhat mutually exclusive with *IDH* mutant tumors, which nonetheless highly express *PDGFRA* [85, 86]*.* Flavanhan et al. initially found *IDH* mutant gliomas exhibited a disrupted CTCF boundary that allowed an aberrant interaction between a typical enhancer and the glioma oncogene *PDGFRA,* each originally insulated from each other, as an alternative method of oncogene activation (Fig. 5) [87]*.*

The same phenomenon occurs in succinate dehydrogenase (*SDH*) silenced tumors [88–90]. In another study, Flavahan et al. showed *SDH* silenced gastrointestinal stromal tumors (GISTs) lose ~ 5% of their CTCF binding sites due to *SDH-*deficient hypermethylation [91]. Under physiologic conditions, one such CTCF site restrains a SE in one TAD from interacting with oncogenes *FGF3* and *FGF4* in the neighboring TAD, while another CTCF site isolates a SE from the GIST oncogene *KIT* [91]*.* CTCF binding loss subsequently leads to aberrant interactions and subsequent oncogene addiction. Aside from methylation, deletions may also inactivate CTCF insulators. Non-coding micro-deletions of TAD boundaries have been described in T-ALL, which merge *TAL1* into an adjacent neighborhood, where it is subsequently activated by enhancers [92].

Whereas we previously discussed examples of TAD boundaries restraining oncogene expression, certain regulatory elements within TADs are also important modulators. Breaking a promoter–enhancer interaction can cause the enhancer to then re-target to another gene promoter in the same neighborhood to form a new, aberrant interaction [93]. A CRISPR interference (CRISPRi) screen of non-coding regions near the *MYC* locus found the promoter of nearby *PVT1,* which is subject to structural rearrangements in cancer, to be a non-coding tumor suppressor element [94]. HiChIP experiments, which capture DNA conformation and contacts, showed that the promoter of *PVT1* sequesters interactions from four nearby enhancers in the same TAD as *MYC.* Upon CRISPRi of the *PVT1* promoter, these enhancers instead interact with the promoter of *MYC,* resulting in increased expression and proliferation [94]. The *PVT1* promoter and surrounding region is observed to be structurally rearranged in some cancers, suggesting these epigenetic changes may undergo positive selection [94]. A limitation of Hi-C experiments has been a lack of resolution, best at defining TADs on the scale of hundreds of kilobases or more. Newer technologies, such as Micro Capture C, have allowed near base pair resolution of promoter-enhancer interactions previously not appreciated using Hi-C techniques. We anticipate these technical advances to further elucidate the evolving relationship of SEs and enhancers to target genes during tumorigenesis. 

### Non-coding translocations

A recent large scale cataloguing effort of non-coding structural variants identified other recurrent alterations near known oncogenes and SEs. At the *MYC* locus, Xu et al. described intra-TAD re-arrangements that shuffle local enhancer regions, which resulted in increased expression and poorer patient outcomes, albeit with a small sample size [95]. Interestingly, only *MYC* expression was sensitive to these local structural variants compared to neighboring genes via mechanisms that remain to be fully elucidated.

Non-coding regions containing SEs and typical enhancers are observed to be translocated, often over large distances in *cis* or in *trans,* to activate nearby proto-oncogenes in a phenomenon that has been termed enhancer hijacking [96, 97]. Acute myeloid leukemia (AML) demonstrating inv(3) or t(3;3) translocations aberrantly place a portion of a *GATA2* SE near the proto-oncogene *EVI1* to drive tumorigenesis [98, 99]. Furthermore, excision of this translocated SE portion resulted in decreased oncogene expression, differentiation, and growth inhibition [98]. In adenoid cystic carcinoma, Gillespie et al. observed translocation of SEs to activate the oncogenic transcription factor *MYB* resulting in increased expression. Interestingly, activated *MYB* forms a feed-forward loop to bind to the translocated SE itself [100]. In diffuse large B cell lymphoma, the SE at the *BCL6* locus similarly serves as an “enhancer donor” in translocations to activate distal oncogenes, such as *MYC* [101]*.*

Conversely, protein-coding regions of proto-oncogenes may be translocated to the vicinity of powerful SEs which lead to their activation. Northcott et al. described *GFI1* and *GFI1B* proto-oncogene translocations into new neighborhoods to hijack endogenous SEs in medulloblastoma [102]. Similarly, Peifer et al. describe the translocation of *TERT* towards strong enhancer elements to facilitate aberrant activation in high-risk neuroblastoma [103]. Finally, Montefiori et al. describe the translocation of the *BCL11B* proto-oncogene to hijack SEs in hematopoietic progenitor cells to drive lineage–ambiguous leukemia [104]. Taken together, these observations in several cancers show recurrent displacement of proto-oncogenes to SEs or SEs to proto-oncogenes to facilitate aberrant transcriptional activation towards tumorigenesis.

### Cell-extrinsic SE reprogramming: in response to microenvironment

Cell extrinsic SE reprogramming occurs in response to hormones, such as estrogen and testosterone [56, 105–109]. In estrogen receptor-positive (ER+) breast cancer, ChIP-seq shows estrogen receptor (ERα) enrichment at gained SEs in breast cancer over normal breast, including at the *ESR1* SE itself which encodes ERα in an autoloop characteristic of SEs (Fig. 1) [7]. However, few studies have examined cell-extrinsic enhancer and SE reprogramming in non-hormonal cancers [56, 105–109]. A study using hair follicle stem cells demonstrated that the local microenvironment influences the SE landscape of hair follicle stem cells (HFSCs) [110]. Only ~10% of SEs were shared between HFSCs grown *in vitro* or *in vivo,* with 36% specific to the *in vivo* setting and ~ 54% specific *in vitro* [110]*.* As proof-of-principle, certain SEs absent in vitro are restored when re-introduced back into their microenvironment *in vivo* [110]. The enhancer landscape of tissue-resident macrophages was also demonstrated to be shaped by their microenvironment as demonstrated through similar, elegant transplantation experiments [111, 112].

**Fig. 6:**
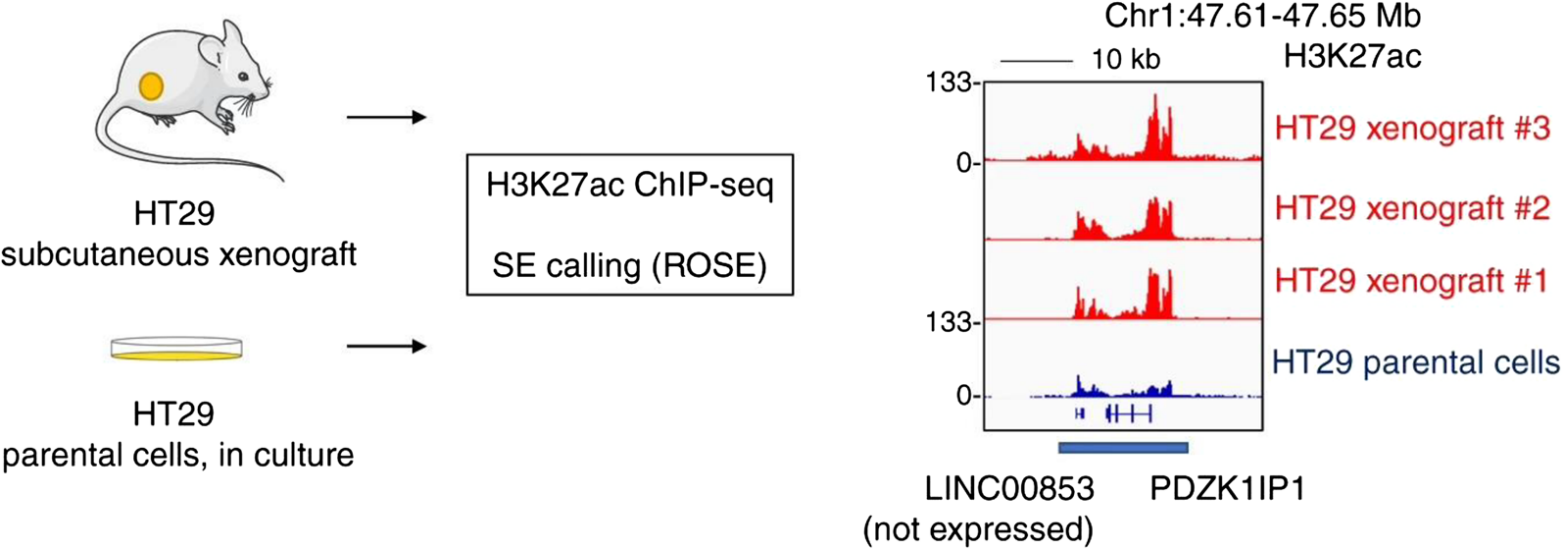
Super-enhancer reprogramming by the local tumor microenvironment. The regulatory region at the locus of *PDZK1IP1* does not meet super-enhancer calling criteria in HT29 cells grown as culture. It exists as a typical enhancer, with minimal expression (~ 2 TPM by RNA-seq). When the same cells are grown as a subcutaneous xenograft on the back of an immunocompromised mouse, the super-enhancer is induced with significant increases in H3K27ac deposition at the region and approximately 30–40 × increase in *PDZK1IP1* expression. Figure reproduced with permission from Nature Communications. Please refer to the original publication (Zhou et al. [114]) for more details and citation

In cancer cells, the first suggestion came when a principal component analysis of H3K27ac signal at enhancers was found to differ between a panel of medulloblastoma primary tumors versus cell lines despite genetic similarities between the two groups [113]. A recent study comparing the SE landscape between a panel of primary CRC tumors against CRC cell lines identified ~ 10% of SEs are unique to the primary tumor setting. Interestingly, this included one of the most recurrently gained SEs in CRC over patient-matched adjacent normal, suggesting cell-extrinsic mechanisms may reprogram key SEs during tumorigenesis [114]. As proof-of-principle, growing the HT29 CRC cell line as a xenograft resulted in the induction of this highly recurrently gained SE (Fig. 6) [114]. Its target, *PDZK1IP1,* was found to be a context-specific dependency gene in CRC—in culture, it is largely silenced without its SE; in xenografts, the SE results in a >30-fold increase in expression that confers metabolic fitness against the oxidative stress environment of the local tumor microenvironment [114].

TF motif analysis at open chromatin regions within this SE implicated inflammation, including STAT1, STAT3, and NF-κB. In line with this observation, treatment of HT29 cells in vitro with the cytokines IL-6, TNFα, and IFN-γ phenocopied the SE induction seen in xenografts. This finding was supported by a previous study, where inflammation and fibrosis were found to drive SE formation in endothelial cells and cardiomyocytes via NF-κB [115, 116].

Other links between inflammation and the cancer epigenome have been noted. Inflammation is crucial to establishing an enhancer network to promote *KRAS* mutant pancreatic tumorigenesis [117, 118]. Another study showed the cytokine interleukin-6 and its downstream transcription factor STAT3 could engage estrogen receptor enhancers in breast cancer metastasis [119]. Furthermore, a recent study demonstrated that clear cell renal cell carcinoma expresses chemokines and cytokines to shape their immune landscape in a process known as cancer-cell-intrinsic inflammation that occurs through SEs gained after serial orthotopic inoculation and lung metastasis [120, 121]. Taken together, these findings highlight the relevant interplay between environment and SEs in cancer.

### Cell-extrinsic SE reprogramming: in response to therapy

There is increasing evidence that the development of resistance against pharmacologic inhibition of oncogenic signaling pathways is associated with SE re-programming that is supported by patient data. For instance, BRAF and MEK inhibition in triple negative breast cancer results in de novo SE formation at receptor tyrosine kinases to promote escape [122]. The MEK inhibitor trametinib induced increased BRD4, H3K27ac, p300, and MED1 at select enhancers and SEs enriched for the motifs of the transcription factors CEBPB and CEBPD [122]. Time course experiments reveal rapid kinetics: BRD4 recruitment at *de novo* enhancers was greatest within 1–4 h of trametinib treatment [122]. Increased occupancy of these transcription factors were confirmed at trametinib-induced enhancers by ChIP-seq. Interestingly, the authors also demonstrate *MYC* is required for these enhancer changes, as they are attenuated by *MYC* silencing [122]. Combining such kinase and BET inhibition as a subsequent therapeutic strategy has been proposed by groups, including ours [122–124]. Conversely, one study utilizing CRISPR screens towards identifying mediators of BET inhibition resistance converged upon phosphoinositide 3-kinase (PI3K) signaling activation, which was accompanied by enhancer reprogramming [124].

Similarly, CDK4/6 inhibition of breast cancer results in SE formation to promote luminal differentiation and evasion of apoptosis [125]. Mechanistically, CDK4/6 inhibition lead to the upregulation of AP-1 complex members *FOS* and *JUN,* in an Rb-dependent manner, which drove these epigenetic changes. The AP-1 complex was previously implicated in the maintenance of a mature luminal mammary cell state [126, 127]. Furthermore, pharmacologic inhibition of AP-1 members reversed the observed enhancer changes [125].

Resistance to BCL-2 inhibitors in mantle cell and double-hit lymphomas are associated with SE reprogramming that causes resistance to these therapies [128]. These resistant cells exhibit loss of 18q21 *BCL2* amplicon [128]. Combining epigenomic profiling with a chemical screen, Zhao et al. found SE reprogramming associated with resistance to BCL-2 inhibitors to be dependent on CDK7 [128]. They identified CDK7 as a synthetic lethality in the setting of BCL-2 inhibitor resistance, and pharmacologic inhibition of CDK7 reversed SE changes associated with BCL-2 inhibitor resistance [128].

As with most of these studies, the exact signaling mechanisms that converge on the epigenome to cause SE reprogramming need higher resolution dissection in future studies. Importantly, these de novo therapy induced SEs involved in *resistance* and *escape* should not be confused with de novo SEs that are seen with *response* to therapy, as seen in MEK/ERK inhibition in Ras-driven rhabdomyosarcomas [129].

## Conclusion

Since the initial identification of SEs decades ago, a great deal of evidence has been generated supporting the concept that SEs play an important role in the development of many types of cancer. As summarized in this review, pathogenic super enhancers can form as the result of genetic changes directly at *cis* or *trans* elements of the transcriptional machinery or can form indirectly as a result of activation of a signaling pathway that ultimately activates the formation of SEs at target genes. The most common deliverable from the study of oncogenic SEs has been the identification of transcriptional dependency genes, including in settings, where recurrent mutations are scarce [130–135]. Upstream of SEs, algorithms such as Coltron can be used to define core transcription factor circuitry that occupy and sustain SEs, often critically essential themselves [136–139]. Analyses of SE landscapes have also been used to subtype and prognosticate individual cancers and discern intra-tumoral cancer cell types [113, 140–148].

Early efforts to selectively target SE expression were aimed at general transcriptional machinery, such as bromodomain-containing proteins, such as BRD4 and CDK7/9/12/13, with the idea that the disproportionate occupancy at SEs would be preferentially depleted as compared to other, physiologic typical enhancer loci [2, 54, 132, 149–152]. Alternatively, a proteolysis-targeting chimera (PROTAC) degrader of the SWI/SNF chromatin remodeling complex ATPase subunits SMARCA2 and SMARCA4 was recently observed to deplete H3K27ac at several oncogenic SEs, including at *MYC, AR, ERG,* and *FOXA1,* resulting in potent suppression of prostate cancer growth in xenograft models [153].

**Fig. 7:**
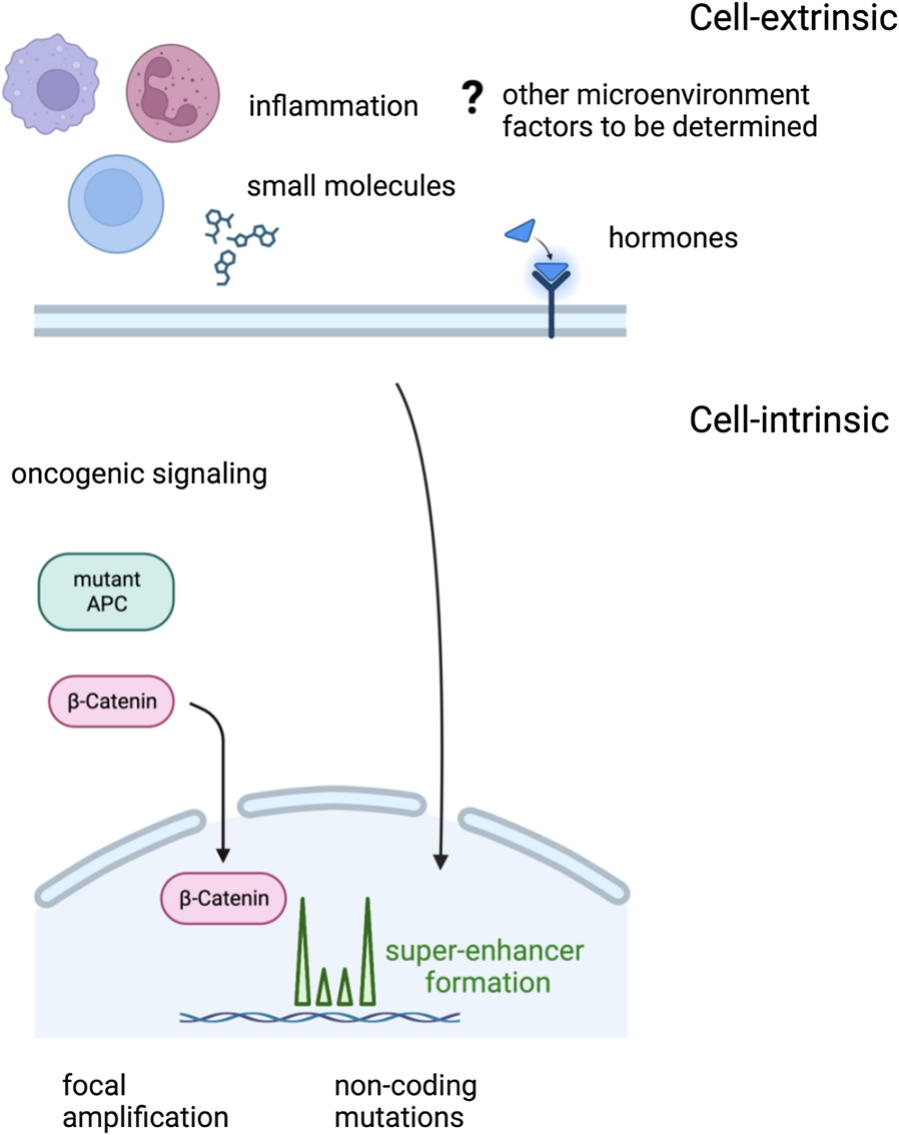
Cell-intrinsic and cell-extrinsic etiologies converge on the epigenome to shape super-enhancer formation in cancer. The roles of genomic alterations, including somatic mutations in cancer drivers and non-coding regions, subsequent oncogenic signaling, as well as focal amplification are well-described mechanisms of super-enhancer activation in cancer. The earliest descriptions of cell-extrinsic enhancer reprogramming were in androgen- and estrogen-dependent cancers, where these hormones would signal to the nucleus. Recently, broader cell-extrinsic etiologies, including super-enhancer reprogramming by inflammation within the local tumor microenvironment and in response to targeted therapies to mediate resistance, have been increasingly described

The most recent advances in understanding the enhancer, SE, and open chromatin landscape of cancer is a greater appreciation that it is shaped by forces within the cell—such as genomic alterations—but also independently by forces outside the cell as well (Fig. 7).

Thus, our wealth of ChIP-seq and ATAC-seq data on primary tumors actually reflect three, distinct super-imposed chromatin landscapes: cell-intrinsic, cell-extrinsic, and a unique third subset that arises from the interplay between the latter two [154]. Indeed, the tumor-microenvironment and inflammation mediated SE at *PDZK1IP1* may fall into this latter category—ubiquitous in primary CRC, yet rare in CRC cell lines (purely cell-intrinsic) as well as inflamed non-dysplastic colon (purely cell-extrinsic) [114]. In particular, how cell-intrinsic and cell-extrinsic forces constrain and synergize with each other to shape the enhancer landscape of cancer will be an exciting area of future study. This area’s relevance is perhaps best exemplified in pancreatic tumorigenesis, where inflammation from local pancreas tissue injury induces a chromatin switch (cell-extrinsic) that cooperates with *Kras* mutations to accelerate lesion formation [154].

Cell-extrinsic enhancer reprogramming may also provide insight towards targeting these oncogenic epigenetic changes. Given the role of inflammation towards their induction, future studies should elucidate whether tamping down local inflammation, using FDA-approved TNF-α inhibitors or PROTAC-degraders of STAT3, could attenuate such reprogramming with therapeutic benefit [155]. While most current studies examining cell-extrinsic forces focused on chromatin, it may be interesting to see whether hypoxia or other extracellular stressors exert long-lasting effects on the epigenome.

## Data Availability

Figure 1 is adapted, with permission from the publisher, from: Hnisz et al. [5]. Figure 2 is adapted, with permission from the publisher, from: Sabari et al*.* [40]. Figure 3 is adapted, with permission from the publisher, from: Mansour et al*.* [8]. Figure 4 is adapted, with permission from the publisher, from: Zhang et al*.* [9]. Figure 5 is adapted, with permission from the publisher, from: Flavahan et al*.* [91]. Figure 6 is adapted, with permission from the publisher, from: Zhou et al*.* [114]. Figure 7 is adapted using the “Wnt Beta-Catenin Signaling Pathway” template from BioRender.com (2023). Retrieved from https://app.biorender.com/biorender-templates. This article is licensed under a Creative Commons Attribution 4.0 International License, which permits use, sharing, and adaptation, distribution and reproduction in any medium or format, as long as the original author(s) are credited. A copy of the license can be found here: https://creativecommons.org/licenses/by/4.0/

## Abbreviations

SE: Super enhancer
ecDNA: Extrachromosomal DNA
PROTAC: Proteolysis-targeting chimera
TAD: Topologically associating domain
ROSE: Rank Ordering of Super-Enhancers
ChIP-seq: Chromatin immunoprecipitation followed by next-generation sequencing
HFSCs: Hair follicle stem cells
ER+: Estrogen receptor positive
AML: Acute myeloid leukemia
CRISPR: Clustered regularly interspaced short palindromic repeats
CRISPRi: CRISPR interference
GISTs: Gastrointestinal stromal tumors
2-HG: 2-Hydroxyglutarte
FISH: Fluorescence in situ hybridization
DLBCL: Diffuse large B cell lymphoma
AID: Activation-induced cytidine deaminase
TEs: Typical enhancers
RCC: Renal cell carcinoma
mESCs: Murine embryonic stem cells

## References

[1] Whyte WA (2013). Master transcription factors and mediator establish super-enhancers at key cell identity genes. Cell.

[2] Loven J (2013). Selective inhibition of tumor oncogenes by disruption of super-enhancers. Cell.

[3] Hnisz D, Shrinivas K, Young RA, Chakraborty AK, Sharp PA (2017). A phase separation model for transcriptional control. Cell.

[4] Prager BC (2020). The meningioma enhancer landscape delineates novel subgroups and drives druggable dependencies. Cancer Discov.

[5] Hnisz D (2013). Super-enhancers in the control of cell identity and disease. Cell.

[6] Bahr C (2018). A Myc enhancer cluster regulates normal and leukaemic haematopoietic stem cell hierarchies. Nature.

[7] Hnisz D (2015). Convergence of developmental and oncogenic signaling pathways at transcriptional super-enhancers. Mol Cell.

[8] Mansour MR (2014). Oncogene regulation. An oncogenic super-enhancer formed through somatic mutation of a noncoding intergenic element. Science.

[9] Zhang X (2016). Identification of focally amplified lineage-specific super-enhancers in human epithelial cancers. Nat Genet.

[10] Morton AR (2019). Functional enhancers shape extrachromosomal oncogene amplifications. Cell.

[11] Yao X (2017). VHL deficiency drives enhancer activation of oncogenes in clear cell renal cell carcinoma. Cancer Discov.

[12] Cancer Genome Atlas N (2012). Comprehensive molecular characterization of human colon and rectal cancer. Nature.

[13] Stamos JL, Weis WI (2013). The beta-catenin destruction complex. Cold Spring Harb Perspect Biol.

[14] Zhang X (2018). Somatic superenhancer duplications and hotspot mutations lead to oncogenic activation of the KLF5 transcription factor. Cancer Discov.

[15] Ying CY (2013). MEF2B mutations lead to deregulated expression of the oncogene BCL6 in diffuse large B cell lymphoma. Nat Immunol.

[16] Brescia P (2018). MEF2B instructs germinal center development and acts as an oncogene in B cell lymphomagenesis. Cancer Cell.

[17] Dhar SS (2018). MLL4 is required to maintain broad H3K4me3 peaks and super-enhancers at tumor suppressor genes. Mol Cell.

[18] Alam H (2020). KMT2D deficiency impairs super-enhancers to confer a glycolytic vulnerability in lung cancer. Cancer Cell.

[19] Sun Y (2018). HOXA9 reprograms the enhancer landscape to promote leukemogenesis. Cancer Cell.

[20] Mathur R (2017). ARID1A loss impairs enhancer-mediated gene regulation and drives colon cancer in mice. Nat Genet.

[21] Schoenfeld DA (2022). Loss of PBRM1 alters promoter histone modifications and activates ALDH1A1 to drive renal cell carcinoma. Mol Cancer Res.

[22] Alver BH (2017). The SWI/SNF chromatin remodelling complex is required for maintenance of lineage specific enhancers. Nat Commun.

[23] Wang X (2017). SMARCB1-mediated SWI/SNF complex function is essential for enhancer regulation. Nat Genet.

[24] Nakayama RT (2017). SMARCB1 is required for widespread BAF complex-mediated activation of enhancers and bivalent promoters. Nat Genet.

[25] Wilson MR (2020). ARID1A mutations promote P300-dependent endometrial invasion through super-enhancer hyperacetylation. Cell Rep.

[26] Reske JJ (2022). ARID1A-dependent maintenance of H3.3 is required for repressive CHD4-ZMYND8 chromatin interactions at super-enhancers. BMC Biol.

[27] Gryder BE (2017). PAX3-FOXO1 establishes myogenic super enhancers and confers BET bromodomain vulnerability. Cancer Discov.

[28] Arabzade A (2021). ZFTA-RELA dictates oncogenic transcriptional programs to drive aggressive supratentorial ependymoma. Cancer Discov.

[29] Huang Y (2019). The leukemogenic TCF3-HLF complex rewires enhancers driving cellular identity and self-renewal conferring EP300 vulnerability. Cancer Cell.

[30] Thirant C (2017). ETO2-GLIS2 hijacks transcriptional complexes to drive cellular identity and self-renewal in pediatric acute megakaryoblastic leukemia. Cancer Cell.

[31] Sin-Chan P (2019). A C19MC-LIN28A-MYCN oncogenic circuit driven by hijacked super-enhancers is a distinct therapeutic vulnerability in etmrs: a lethal brain tumor. Cancer Cell.

[32] Han TW (2012). Cell-free formation of RNA granules: bound RNAs identify features and components of cellular assemblies. Cell.

[33] Li P (2012). Phase transitions in the assembly of multivalent signalling proteins. Nature.

[34] Lin Y, Protter DS, Rosen MK, Parker R (2015). Formation and maturation of phase-separated liquid droplets by RNA-binding proteins. Mol Cell.

[35] Boija A (2018). Transcription factors activate genes through the phase-separation capacity of their activation domains. Cell.

[36] Shrinivas K (2019). Enhancer features that drive formation of transcriptional condensates. Mol Cell.

[37] Cho WK (2018). Mediator and RNA polymerase II clusters associate in transcription-dependent condensates. Science.

[38] Chong S (2018). Imaging dynamic and selective low-complexity domain interactions that control gene transcription. Science.

[39] Cai D (2019). Phase separation of YAP reorganizes genome topology for long-term YAP target gene expression. Nat Cell Biol.

[40] Sabari BR (2018). Coactivator condensation at super-enhancers links phase separation and gene control. Science.

[41] Lyons H (2023). Functional partitioning of transcriptional regulators by patterned charge blocks. Cell.

[42] Boija A, Klein IA, Young RA (2021). Biomolecular condensates and cancer. Cancer Cell.

[43] Klein IA (2020). Partitioning of cancer therapeutics in nuclear condensates. Science.

[44] Ahn JH (2021). Phase separation drives aberrant chromatin looping and cancer development. Nature.

[45] Chandra B (2022). Phase separation mediates NUP98 fusion oncoprotein leukemic transformation. Cancer Discov.

[46] Meng FL (2014). Convergent transcription at intragenic super-enhancers targets AID-initiated genomic instability. Cell.

[47] Qian J (2014). B cell super-enhancers and regulatory clusters recruit AID tumorigenic activity. Cell.

[48] Bal E (2022). Super-enhancer hypermutation alters oncogene expression in B cell lymphoma. Nature.

[49] Shen JC (2019). A high-resolution landscape of mutations in the BCL6 super-enhancer in normal human B cells. Proc Natl Acad Sci USA.

[50] Oldridge DA (2015). Genetic predisposition to neuroblastoma mediated by a LMO1 super-enhancer polymorphism. Nature.

[51] Li Z (2017). APOBEC signature mutation generates an oncogenic enhancer that drives LMO1 expression in T-ALL. Leukemia.

[52] Huang M, Weiss WA (2013). Neuroblastoma and MYCN. Cold Spring Harb Perspect Med.

[53] Zeid R (2018). Enhancer invasion shapes MYCN-dependent transcriptional amplification in neuroblastoma. Nat Genet.

[54] Chipumuro E (2014). CDK7 inhibition suppresses super-enhancer-linked oncogenic transcription in MYCN-driven cancer. Cell.

[55] Takeda DY (2018). A somatically acquired enhancer of the androgen receptor is a noncoding driver in advanced prostate cancer. Cell.

[56] Porter LH (2021). Androgen receptor enhancer amplification in matched patient-derived xenografts of primary and castrate-resistant prostate cancer. J Pathol.

[57] Glodzik D (2017). A somatic-mutational process recurrently duplicates germline susceptibility loci and tissue-specific super-enhancers in breast cancers. Nat Genet.

[58] Herranz D (2014). A NOTCH1-driven MYC enhancer promotes T cell development, transformation and acute lymphoblastic leukemia. Nat Med.

[59] Zimmerman MW (2018). MYC drives a subset of high-risk pediatric neuroblastomas and is activated through mechanisms including enhancer hijacking and focal enhancer amplification. Cancer Discov.

[60] Corces MR (2018). The chromatin accessibility landscape of primary human cancers. Science.

[61] Shoshani O (2021). Chromothripsis drives the evolution of gene amplification in cancer. Nature.

[62] Rosswog C (2021). Chromothripsis followed by circular recombination drives oncogene amplification in human cancer. Nat Genet.

[63] Nathanson DA (2014). Targeted therapy resistance mediated by dynamic regulation of extrachromosomal mutant EGFR DNA. Science.

[64] Rausch T (2012). Genome sequencing of pediatric medulloblastoma links catastrophic DNA rearrangements with TP53 mutations. Cell.

[65] Kim H (2020). Extrachromosomal DNA is associated with oncogene amplification and poor outcome across multiple cancers. Nat Genet.

[66] Yi E (2022). Live-cell imaging shows uneven segregation of extrachromosomal DNA elements and transcriptionally active extrachromosomal DNA hubs in cancer. Cancer Discov.

[67] Lange JT (2022). The evolutionary dynamics of extrachromosomal DNA in human cancers. Nat Genet.

[68] Wu S (2019). Circular ecDNA promotes accessible chromatin and high oncogene expression. Nature.

[69] Korbel JO, Campbell PJ (2013). Criteria for inference of chromothripsis in cancer genomes. Cell.

[70] Helmsauer K (2020). Enhancer hijacking determines extrachromosomal circular MYCN amplicon architecture in neuroblastoma. Nat Commun.

[71] Zhu Y (2021). Oncogenic extrachromosomal DNA functions as mobile enhancers to globally amplify chromosomal transcription. Cancer Cell.

[72] Oobatake Y, Shimizu N (2020). Double-strand breakage in the extrachromosomal double minutes triggers their aggregation in the nucleus, micronucleation, and morphological transformation. Genes Chromosomes Cancer.

[73] Hung KL (2021). ecDNA hubs drive cooperative intermolecular oncogene expression. Nature.

[74] Hung KL (2022). Targeted profiling of human extrachromosomal DNA by CRISPR-CATCH. Nat Genet.

[75] Gong Y (2018). Stratification of TAD boundaries reveals preferential insulation of super-enhancers by strong boundaries. Nat Commun.

[76] Beagrie RA (2017). Complex multi-enhancer contacts captured by genome architecture mapping. Nature.

[77] Guo Y (2015). CRISPR inversion of CTCF sites alters genome topology and enhancer/promoter function. Cell.

[78] Lupianez DG (2015). Disruptions of topological chromatin domains cause pathogenic rewiring of gene-enhancer interactions. Cell.

[79] Nora EP (2017). Targeted degradation of CTCF decouples local insulation of chromosome domains from genomic compartmentalization. Cell.

[80] Hark AT (2000). CTCF mediates methylation-sensitive enhancer-blocking activity at the H19/Igf2 locus. Nature.

[81] Liu XS (2016). Editing DNA methylation in the mammalian genome. Cell.

[82] Bell AC, Felsenfeld G (2000). Methylation of a CTCF-dependent boundary controls imprinted expression of the Igf2 gene. Nature.

[83] Kaelin WG, McKnight SL (2013). Influence of metabolism on epigenetics and disease. Cell.

[84] Dang L, Yen K, Attar EC (2016). IDH mutations in cancer and progress toward development of targeted therapeutics. Ann Oncol.

[85] Verhaak RG (2010). Integrated genomic analysis identifies clinically relevant subtypes of glioblastoma characterized by abnormalities in PDGFRA, IDH1, EGFR, and NF1. Cancer Cell.

[86] Brennan CW (2013). The somatic genomic landscape of glioblastoma. Cell.

[87] Flavahan WA (2016). Insulator dysfunction and oncogene activation in IDH mutant gliomas. Nature.

[88] Killian JK (2013). Succinate dehydrogenase mutation underlies global epigenomic divergence in gastrointestinal stromal tumor. Cancer Discov.

[89] Janeway KA (2011). Defects in succinate dehydrogenase in gastrointestinal stromal tumors lacking KIT and PDGFRA mutations. Proc Natl Acad Sci USA.

[90] Xiao M (2012). Inhibition of alpha-KG-dependent histone and DNA demethylases by fumarate and succinate that are accumulated in mutations of FH and SDH tumor suppressors. Genes Dev.

[91] Flavahan WA (2019). Altered chromosomal topology drives oncogenic programs in SDH-deficient GISTs. Nature.

[92] Hnisz D (2016). Activation of proto-oncogenes by disruption of chromosome neighborhoods. Science.

[93] Oh S (2021). Enhancer release and retargeting activates disease-susceptibility genes. Nature.

[94] Cho SW (2018). Promoter of lncRNA Gene PVT1 is a tumor-suppressor DNA boundary element. Cell.

[95] Xu Z (2022). Structural variants drive context-dependent oncogene activation in cancer. Nature.

[96] Wang X (2021). Genome-wide detection of enhancer-hijacking events from chromatin interaction data in rearranged genomes. Nat Methods.

[97] Weischenfeldt J (2017). Pan-cancer analysis of somatic copy-number alterations implicates IRS4 and IGF2 in enhancer hijacking. Nat Genet.

[98] Groschel S (2014). A single oncogenic enhancer rearrangement causes concomitant EVI1 and GATA2 deregulation in leukemia. Cell.

[99] Smeenk L (2021). Selective requirement of MYB for oncogenic hyperactivation of a translocated enhancer in leukemia. Cancer Discov.

[100] Drier Y (2016). An oncogenic MYB feedback loop drives alternate cell fates in adenoid cystic carcinoma. Nat Genet.

[101] Ryan RJ (2015). Detection of enhancer-associated rearrangements reveals mechanisms of oncogene dysregulation in B-cell lymphoma. Cancer Discov.

[102] Northcott PA (2014). Enhancer hijacking activates GFI1 family oncogenes in medulloblastoma. Nature.

[103] Peifer M (2015). Telomerase activation by genomic rearrangements in high-risk neuroblastoma. Nature.

[104] Montefiori LE (2021). Enhancer hijacking drives oncogenic BCL11B expression in lineage-ambiguous stem cell leukemia. Cancer Discov.

[105] Wang Q (2009). Androgen receptor regulates a distinct transcription program in androgen-independent prostate cancer. Cell.

[106] Fullwood MJ (2009). An oestrogen-receptor-alpha-bound human chromatin interactome. Nature.

[107] Hurtado A, Holmes KA, Ross-Innes CS, Schmidt D, Carroll JS (2011). FOXA1 is a key determinant of estrogen receptor function and endocrine response. Nat Genet.

[108] Hamilton WB (2019). Dynamic lineage priming is driven via direct enhancer regulation by ERK. Nature.

[109] Cavalli G, Heard E (2019). Advances in epigenetics link genetics to the environment and disease. Nature.

[110] Adam RC (2015). Pioneer factors govern super-enhancer dynamics in stem cell plasticity and lineage choice. Nature.

[111] Lavin Y (2014). Tissue-resident macrophage enhancer landscapes are shaped by the local microenvironment. Cell.

[112] Gosselin D (2014). Environment drives selection and function of enhancers controlling tissue-specific macrophage identities. Cell.

[113] Lin CY (2016). Active medulloblastoma enhancers reveal subgroup-specific cellular origins. Nature.

[114] Zhou RW (2022). A local tumor microenvironment acquired super-enhancer induces an oncogenic driver in colorectal carcinoma. Nat Commun.

[115] Brown JD (2014). NF-kappaB directs dynamic super enhancer formation in inflammation and atherogenesis. Mol Cell.

[116] Duan Q (2017). BET bromodomain inhibition suppresses innate inflammatory and profibrotic transcriptional networks in heart failure. Sci Transl Med.

[117] Li Y (2021). Mutant Kras co-opts a proto-oncogenic enhancer network in inflammation-induced metaplastic progenitor cells to initiate pancreatic cancer. Nat Cancer.

[118] Alonso-Curbelo D (2021). A gene-environment-induced epigenetic program initiates tumorigenesis. Nature.

[119] Siersbaek R (2020). IL6/STAT3 signaling hijacks estrogen receptor alpha enhancers to drive breast cancer metastasis. Cancer Cell.

[120] Nishida J (2020). Epigenetic remodelling shapes inflammatory renal cancer and neutrophil-dependent metastasis. Nat Cell Biol.

[121] Rodrigues P (2018). NF-kappaB-dependent lymphoid enhancer co-option promotes renal carcinoma metastasis. Cancer Discov.

[122] Zawistowski JS (2017). Enhancer remodeling during adaptive bypass to mek inhibition is attenuated by pharmacologic targeting of the P-TEFb complex. Cancer Discov.

[123] Stratikopoulos EE (2015). Kinase and BET inhibitors together clamp inhibition of PI3K signaling and overcome resistance to therapy. Cancer Cell.

[124] Iniguez AB (2018). Resistance to epigenetic-targeted therapy engenders tumor cell vulnerabilities associated with enhancer remodeling. Cancer Cell.

[125] Watt AC (2021). CDK4/6 inhibition reprograms the breast cancer enhancer landscape by stimulating AP-1 transcriptional activity. Nat Cancer.

[126] Dravis C (2018). Epigenetic and transcriptomic profiling of mammary gland development and tumor models disclose regulators of cell state plasticity. Cancer Cell.

[127] Pellacani D (2016). Analysis of normal human mammary epigenomes reveals cell-specific active enhancer states and associated transcription factor networks. Cell Rep.

[128] Zhao X (2019). BCL2 amplicon loss and transcriptional remodeling drives ABT-199 resistance in B cell lymphoma models. Cancer Cell.

[129] Yohe ME (2018). MEK inhibition induces MYOG and remodels super-enhancers in RAS-driven rhabdomyosarcoma. Sci Transl Med.

[130] Chapuy B (2013). Discovery and characterization of super-enhancer-associated dependencies in diffuse large B cell lymphoma. Cancer Cell.

[131] Nagaraja S (2017). Transcriptional dependencies in diffuse intrinsic pontine glioma. Cancer Cell.

[132] Christensen CL (2014). Targeting transcriptional addictions in small cell lung cancer with a covalent CDK7 inhibitor. Cancer Cell.

[133] Mack SC (2018). Therapeutic targeting of ependymoma as informed by oncogenic enhancer profiling. Nature.

[134] Roe JS (2017). Enhancer reprogramming promotes pancreatic cancer metastasis. Cell.

[135] Morrow JJ (2018). Positively selected enhancer elements endow osteosarcoma cells with metastatic competence. Nat Med.

[136] Gryder BE (2019). Histone hyperacetylation disrupts core gene regulatory architecture in rhabdomyosarcoma. Nat Genet.

[137] Durbin AD (2018). Selective gene dependencies in MYCN-amplified neuroblastoma include the core transcriptional regulatory circuitry. Nat Genet.

[138] Sharifnia T (2019). Small-molecule targeting of brachyury transcription factor addiction in chordoma. Nat Med.

[139] Ott CJ (2018). Enhancer architecture and essential core regulatory circuitry of chronic lymphocytic leukemia. Cancer Cell.

[140] McKeown MR (2017). Superenhancer analysis defines novel epigenomic subtypes of non-APL AML, including an RARalpha dependency targetable by SY-1425, a potent and selective RARalpha agonist. Cancer Discov.

[141] Cejas P (2019). Enhancer signatures stratify and predict outcomes of non-functional pancreatic neuroendocrine tumors. Nat Med.

[142] van Groningen T (2017). Neuroblastoma is composed of two super-enhancer-associated differentiation states. Nat Genet.

[143] Boeva V (2017). Heterogeneity of neuroblastoma cell identity defined by transcriptional circuitries. Nat Genet.

[144] Gartlgruber M (2021). Super enhancers define regulatory subtypes and cell identity in neuroblastoma. Nat Cancer.

[145] Tanaka Y (2021). Multi-omic profiling of peritoneal metastases in gastric cancer identifies molecular subtypes and therapeutic vulnerabilities. Nat Cancer.

[146] Prager BC (2020). The meningioma enhancer landscape delineates novel subgroups and drives druggable dependencies. Cancer Discov.

[147] Johann PD (2016). Atypical teratoid/rhabdoid tumors are comprised of three epigenetic subgroups with distinct enhancer landscapes. Cancer Cell.

[148] Garancher A (2018). NRL and CRX define photoreceptor identity and reveal subgroup-specific dependencies in medulloblastoma. Cancer Cell.

[149] Shu S (2016). Response and resistance to BET bromodomain inhibitors in triple-negative breast cancer. Nature.

[150] Asangani IA (2014). Therapeutic targeting of BET bromodomain proteins in castration-resistant prostate cancer. Nature.

[151] Kwiatkowski N (2014). Targeting transcription regulation in cancer with a covalent CDK7 inhibitor. Nature.

[152] Tang Y (2014). Epigenetic targeting of Hedgehog pathway transcriptional output through BET bromodomain inhibition. Nat Med.

[153] Xiao L (2022). Targeting SWI/SNF ATPases in enhancer-addicted prostate cancer. Nature.

[154] Alonso-Curbelo D (2021). A gene-environment-induced epigenetic program initiates tumorigenesis. Nature.

[155] Bai L (2019). A potent and selective small-molecule degrader of STAT3 achieves complete tumor regression in vivo. Cancer Cell.

